# Designing Robust N-of-1 Studies for Precision Medicine: Simulation Study and Design Recommendations

**DOI:** 10.2196/12641

**Published:** 2019-04-01

**Authors:** Bethany Percha, Edward B Baskerville, Matthew Johnson, Joel T Dudley, Noah Zimmerman

**Affiliations:** 1 Icahn School of Medicine at Mount Sinai New York, NY United States

**Keywords:** n-of-1 studies, computer simulation, patient-specific modeling, precision medicine, cross-over studies, inter-individual biological variation, individual differences

## Abstract

**Background:**

Recent advances in molecular biology, sensors, and digital medicine have led to an explosion of products and services for high-resolution monitoring of individual health. The N-of-1 study has emerged as an important methodological tool for harnessing these new data sources, enabling researchers to compare the effectiveness of health interventions at the level of a single individual.

**Objective:**

N-of-1 studies are susceptible to several design flaws. We developed a model that generates realistic data for N-of-1 studies to enable researchers to optimize study designs in advance.

**Methods:**

Our stochastic time-series model simulates an N-of-1 study, incorporating all study-relevant effects, such as carryover and wash-in effects, as well as various sources of noise. The model can be used to produce realistic simulated data for a near-infinite number of N-of-1 study designs, treatment profiles, and patient characteristics.

**Results:**

Using simulation, we demonstrate how the number of treatment blocks, ordering of treatments within blocks, duration of each treatment, and sampling frequency affect our ability to detect true differences in treatment efficacy. We provide a set of recommendations for study designs on the basis of treatment, outcomes, and instrument parameters, and make our simulation software publicly available for use by the precision medicine community.

**Conclusions:**

Simulation can facilitate rapid optimization of N-of-1 study designs and increase the likelihood of study success while minimizing participant burden.

## Introduction

### The Promise of N-of-1 Studies

**Figure 1:**
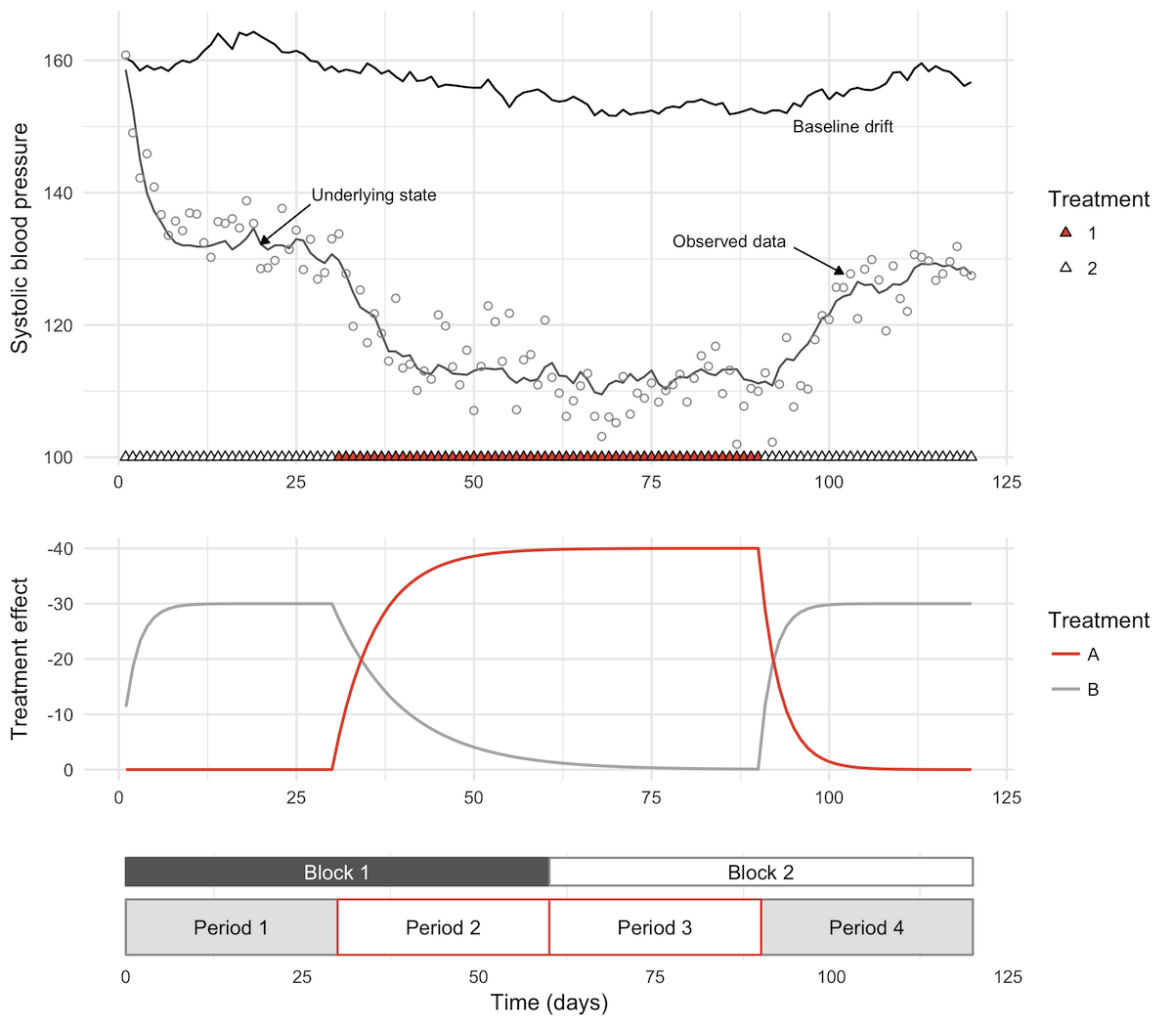
Example of an N-of-1 study comparing two blood pressure medications. An N-of-1 study consists of a set of N blocks, each of which contains J different treatment periods. The order of the treatment periods within each block is usually randomized. Parameters: X0=160, E1=-40, E2=-30, tau1=6.0, gamma1=3.0, tau2=2.0, gamma2=10.0, alpha=0.5, *P*=30, N=2, J=2, sigma_b=0.9, sigma_p=1.0, sigma_0=4.0. In this example, one sample was taken per day.

N-of-1 studies have shown great promise as a tool for investigating the effects of drugs, supplements, behavioral changes, and other health interventions on individual patients [1-7]. An N-of-1 study (Figure 1) is a multiple-crossover comparative effectiveness study of a single patient. Competing treatments are administered in blocks, within which treatment order is randomized or counterbalanced [6]. The outcome of interest is compared across different treatment periods to find the treatment with the greatest efficacy for that specific patient.

N-of-1 studies inform the care of individual patients while simultaneously generating evidence that can be combined with other N-of-1 studies to yield population-level analyses [8-10]. These studies will likely play a key role in precision medicine, with its focus on narrowly defined patient cohorts, rare conditions, and complex comorbidities [5].

### Challenges to N-of-1 Studies

However, the design and analysis of N-of-1 studies present several methodological challenges. Although the Agency for Healthcare Research and Quality has recently released a set of statistical guidelines for N-of-1 studies [6,11], drawing attention to potential treatment effect confounders like underlying time trends, carryover effects, and autocorrelated measurements, there is currently no universal methodological or statistical framework for the design and analysis of N-of-1 trials. Treatments are often compared graphically or ad hoc measures of efficacy are used that differ from study to study; a review of N-of-1 trials published between 1985 and 2010 found that only 49% used any statistical measure to compare treatments [2]. As a result, it is difficult to compare findings from different studies or understand how specific analytic choices influence study results.

N-of-1 studies must also overcome daunting practical and logistical challenges. For example, although researchers might like to administer treatments over dozens of blocks to increase statistical power, such designs are burdensome to the patient and increase the likelihood of attrition. It is also difficult to convince individuals to revisit earlier treatments, especially if these are perceived as less effective [1,6]. Practically speaking, this means the number of treatment blocks in an N-of-1 study is limited, as is the total duration of the study. Although a statistician might prefer more shorter blocks relative to few longer blocks (since the number of samples in a traditional N-of-1 analysis is linear in the number of blocks), rapid switching among treatments may obscure true differences in efficacy because of carryover effects from earlier treatments. Many treatments, such as antidepressants, also take time to display their full effects. Decisions about the length and arrangement of treatment periods can have a profound effect on statistical effect estimates in N-of-1 studies.

### Simulating N-of-1 Studies

Simulation has played a crucial role in clinical trial design, increasing the efficiency and cost-effectiveness of clinical trials, especially in the pharmaceutical industry [12]. Inspired by this, we have developed a stochastic time-series simulation model for N-of-1 studies that incorporates all study-relevant effects, such as carryover and wash-in effects. The model can be used to produce realistic simulated data for a near-infinite number of N-of-1 study designs, treatment profiles, and patient characteristics. The model also incorporates noise parameters like baseline drift, short-term fluctuations (process noise), and measurement error to provide realistic sources of variation that can obscure treatment effects in real-patient settings. Using simulation, we can cheaply and easily investigate how design parameters like sampling frequency, number, and location of samples within blocks, treatment order within blocks, treatment period duration, and total number of blocks impact statistical estimates of treatment effects.

In this paper, we use the model to analyze two N-of-1 case studies, showing how simulation can both optimize study designs and assist researchers in deciding on an appropriate analysis protocol. We then use the model to produce a set of design recommendations for N-of-1 studies on the basis of parameters related to the study outcome, instrument used to measure the outcome, and treatment(s) themselves. We provide our simulation software as a supplement to the paper.

## Methods

### Stochastic Time-Series Model

Assume that there are *J* total treatments in an N-of-1 study. Let *B(t)* denote the patient’s true baseline at time *t*. Let *X*_*j*_*(t)* denote the effect of treatment *j* (*j*=1, …, *J*) at time *t* so that the total treatment effect at time *t* is *X=* Σ_j_ *X*_*j*_*(t)*. Let *T*_*j*_*(t)* be 1 if treatment *j* is in process at time *t* and 0 otherwise (see Figure 1). Let *Z(t)* denote the patient’s true outcome state at time *t*, and let *Y(t)* denote the patient’s observed outcome at time *t*.

The underlying effect driver for each treatment is described as an ordinary differential equation:

dX_j_ = [((E_j_ – X_j_) / *τ*_*j*_) T_j_(t) – (X_j_/ *γ*_j_) (1 – T_j_(t))] dt

Here each *X*_*j*_*(t)* is an exponential decay toward a target value that changes over time—either *E*_*j*_ or 0, depending on *T*_*j*_*(t)* —with time constant *τ*_*j*_ during run-in (decay toward *E*_*j*_) and *γ*_*j*_ during wash-out (decay toward 0).

Baseline drift is simulated as a discretized Wiener process, where normal noise with variance *σ*_*b*_^*2*^Δ *t* is applied every Δ *t*:

B(t + Δt) = B(t) + ΔB(t)

where

ΔB(t) ~ Normal(0, σ_b_^2^ Δt)

The outcome variable *Z(t)* is also a discrete-time stochastic process,

Z(t + Δt) = Z(t) + ΔZ_det_(t) + ΔZ_stoch_(t)

where Δ *Z*_det_*(t)* is a deterministic exponential decay toward the target *X*_*j*_*(t)+B(t)*:

ΔZ_det_(t) = Q(t) + [Z(t) – Q(t)] exp(-Δt/∝)

Q(t) = B(t) + Σ_j_
*X*_*j*_*(t)*

with time constant ∝ and

ΔZ_stoch_(t) ~ Normal(0, σ_p_^2^Δt)

The observed outcome differs from the true outcome only through the addition of normally distributed observation noise:

Y(t) ~ Normal(Z(t), σ_o_)

All of the model parameters are summarized in Table 1. Transformations of *Y(t)* can be used to model different types of outcome parameters, such as scores, counts, and binary outcomes (Table 2).

**Table 1:** The parameters underlying data generation for an N-of-1 study. The parameters are divided into study design parameters (D), treatment-related parameters (T), measurement parameters (M), and outcome-related parameters (O).

Parameter	Type	Description
{t_1_,…,t_n_}	D	Sampling times
N	D	Number of blocks (each with J periods in random order)
J	D	Number of treatment periods per block
P	D	Treatment period length
E_1_,…,E_J_	T	Effect sizes for treatments 1 through J
*τ*_1_,…, *τ*_J_	T	Run-in time constants for treatments 1 through J
*γ*_1_,…, *γ*_J_	T	Wash-out time constants for treatments 1 through J
∝	O	Sensitivity to treatment effect
σ_b_^2^	O	Variance of baseline drift process
σ_p_^2^	O	Variance of process noise
σ_o_^2^	M	Variance of observation noise

**Table 2:** Suggested transformations of *Y* for simulating discrete outcomes.

Outcome type	Range of outcome	Distribution of Y	Transformation
Numeric	Real numbers	—^a^	Identity
Score	[0,…,M]	—	Identity (round, truncate)
Count	[0,…,infinity)	Poisson(λ)	λ = exp(Y)
Proportion	[0,…,M]	Binomial(M, p)	*P*=1/(1 + exp(-Y))
Binary	{0, 1}	Bernoulli(p)	*P*=1/(1 + exp(-Y))

^a^Not applicable.

### Hypertension Case Study

A sample data set and all parameter values for the hypertension case study can be found in Figure 1. The study involves 2 different blood pressure medications, one of which reduces systolic blood pressure by 10 more points than the other in the long run. The more effective medication, treatment 1, takes longer to reach its full effect (*τ*_*1*_=6.0, *τ*_*2*_=2.0) and less time to wash out (*γ*_*1*_=3.0, *γ*_*2*_=10.0). The sampling rate is 1 sample/day, which we chose to model blood pressure that is monitored using a cuff.

We chose a statistical model for this study that incorporated fixed effects for both block ID and treatment, on the basis of the recommendations provided by the Agency for Healthcare Research and Quality (AHRQ) and others [6,11]:

y=β_0_ + β_1_ x_1_ + β_2_ x_2_ + … + β_N_ x_N_

where *x*_*1*_ is 1 if treatment 2 is in progress at the time of the sample, and 0 otherwise, and *x*_*n*_ is 1 if block *n* is in progress, and 0 otherwise. Note that there are only *n−1* indicator variables for blocks; block 1 is used as the reference block. We experimented with other models but found that although modeling choices could affect power, effect size estimates did not change much among models. Our software provides the ability to choose from among several different models.

**Figure 2:**
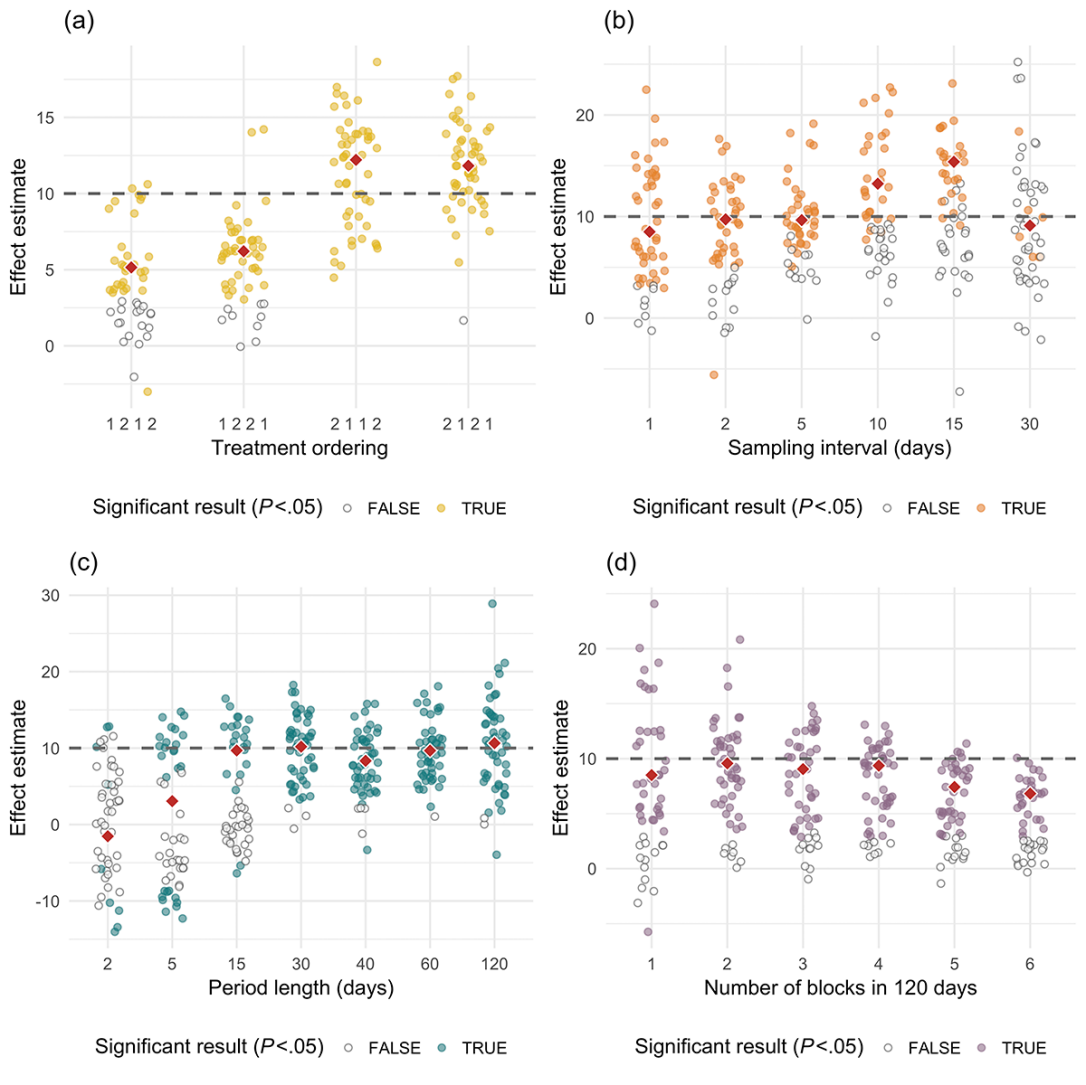
Variation in effect estimates for the hypertension study by study design parameters, including (a) treatment period ordering, (b) sampling frequency, (c) treatment period length, and (d) number of blocks for a fixed study length. The true effect size is 10, illustrated by the dashed lines in the figures. The red diamonds correspond to the median effect size for the statistically significant results within each group. Power estimates were obtained by calculating the ratio of the number of colored dots to the number of total dots. There are 50 trials shown for each parameter setting.

To create Figure 2, we repeated the data generation and analysis process, varying the following parameters and keeping the rest constant:

1. Treatment period orderings were varied among 1 2 1 2, 1 2 2 1, 2 1 1 2, and 2 1 2 1.
2. Sampling frequency was varied from 1 sample per day to 1 sample per treatment period, holding the treatment period ordering fixed at 2 1 2 1.
3. Upon holding sampling frequency constant at 1 sample per day, period length was varied from 2 to 120 days.
4. Study length was held constant at 120 days, and the number of blocks was varied from 1 to 6.

### Pain Management Case Study

The trial design used in this case study emulated the design described in a study by Wegman et al [13]. Although we did not have access to the raw data for this trial and had to estimate reasonable noise parameters and wash-in/wash-out time constants, our goal was simply to compare the analysis technique from the paper with a more traditional approach involving a regression model with fixed effects for treatment and blocks [11]. The regression model we chose was the same as for the first case study.

**Figure 3:**
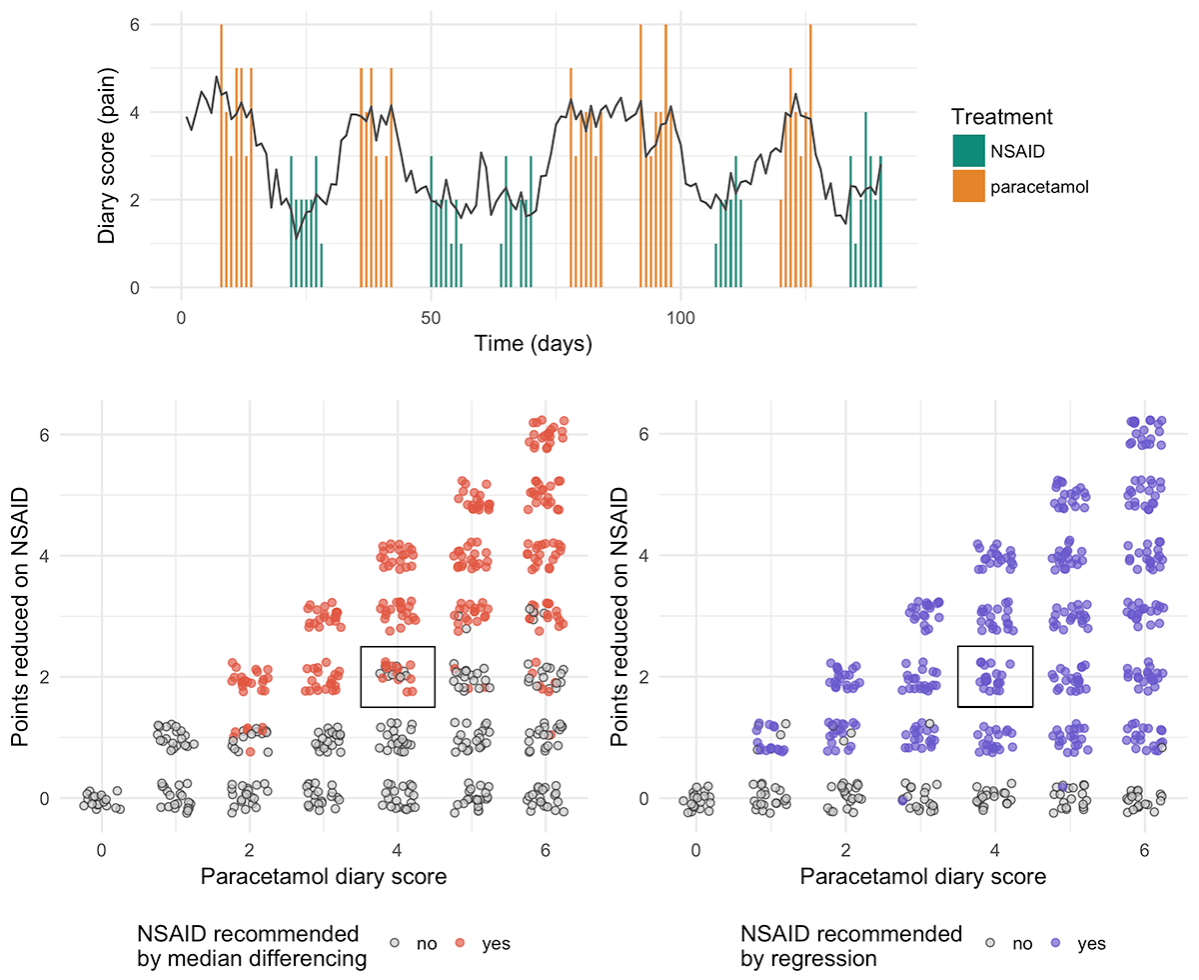
Analyzing a published N-of-1 study comparing NSAIDs to paracetamol. (top) An example simulation in which the true diary score on the NSAID is 2 and on paracetamol is 4. The black line shows the simulated mean outcome (unobserved) at each timepoint, and the colored bars show the observed data, which are discrete scores between 0 and 6. (bottom) A comparison of median differencing, the analysis method described in the paper, with a standard regression model. At the noise levels and effect sizes shown in (top), median differencing will recommend an NSAID only about 60% of the time (black rectangle), whereas a regression model will recommend it 100% of the time. Model parameters: tau1=tau2=1.0 day, gamma1=gamma2=3.5 days, alpha=1.0, sigma_b=0.0 (no baseline drift), sigma_p=0.5, sigma_o=1.0. NSAID: nonsteroidal antiinflammatory drug.

The parameters we chose for this model can be found in Figure 3. We based our decisions about the wash-in and wash-out parameters (*τ* and *γ*) on the fact that the authors chose a wash-out period of 1 week for the different treatments and the fact that both nonsteroidal anti-inflammatory drugs (NSAIDs) and paracetamol are short-acting drugs. We converted the numeric value of the patient state to a discrete score by rounding and truncating it as shown in Table 2.

### Simulations for Design Recommendations

**Figure 4:**
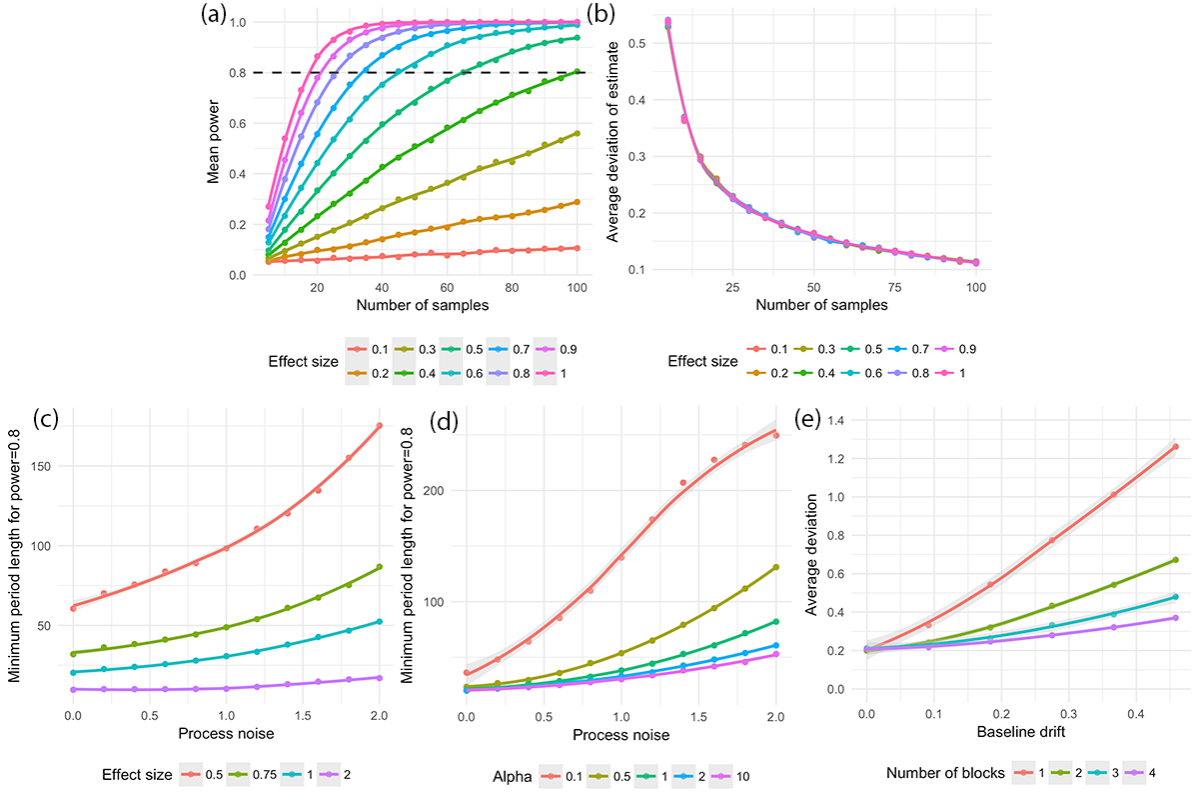
Examining the effect of study design choices on power and accuracy of effect size estimates for an N-of-1 study with effectively instantaneous transitions between treatment states. (a) Effect size vs power for fixed observation noise (sigma_0=1.0) and no process noise or baseline drift. (b) Average deviation of estimate from true value vs. effect size for fixed observation noise (sigma_0=1.0) and no process noise or baseline drift. (c) Minimum treatment period length (ie. number of samples per treatment, with sampling rate fixed at 1 sample per time unit) required to attain a power of 0.8, for varying degrees of process noise and varying effect sizes. No observation noise or baseline drift is present. (d) Same as (c) except effect size is fixed at 1.0 and alpha (individual treatment response) is varied. (e) Average deviation of effect size estimate from its true value, as a function of baseline drift and number of blocks. The effect of baseline drift on the estimate is much more pronounced when fewer blocks are used. **Editorial Notice:** in (a) and (b), x-axis labels should correctly read “Number of samples per treatment.”

All of the simulations in Figure 4 use a baseline of 0 and time constants (*τ*_*1*_*, τ*_*2*_*, γ*_*1*_*,* and *γ*_*2*_) of 0.01. Since treatment 1 is assumed to be placebo, its effect size, *E*_*1*_, is 0. We used a high value for the “sensitivity to treatment effect” parameter (α=10) to produce a near-instantaneous effect. The first and second experiments in Figure 4 used only a single block, as in the absence of any sources of noise except observation noise, block design does not matter. The rest of the parameter choices are outlined in the figure. Each dot represents an average of 50 trials. The smoothed lines shown in Figure 4 are LOESS (LOcally-Estimated Scatterplot Smoothing) fits produced using geom_smooth with default parameters in ggplot, with spans of 0.4, 0.3, 1.0, 1.0, and 1.0 for subfigures a, b, c, d, and e, respectively.

### Data and Code Availability

The simulation software is available in the *n1-simulator* repository under the *HD2i* organization on GitHub. Full details of the available experiments and associated plots are included with the software, along with the data sets generated in the course of making the figures.

## Results

### Modeling the Key Features of an N-of-1 Study

The complete set of parameters for our model can be found in Table 1. The basic model comprises an underlying deterministic process (the growth and decay of treatment effects over time) in addition to 3 types of noise: random baseline drift (eg, long-term illness onset and recovery processes, gaining/losing weight, long-term changes in blood pressure), process noise, which manifests as short-term fluctuations (eg, heart rate and blood pressure volatility, periods of activity/inactivity, and changes in sleep and diet from day to day), and observation noise, which is a function of the instrument and is not related to any underlying biological effect (eg, the measurement noise associated with the cuff that is used to monitor blood pressure).

We divided the parameters into 4 groups: *study design parameters*, which the study designer can vary, *treatment parameters*, which are immutable features of the particular treatments under consideration, a *measurement parameter*, which is a feature of the device used to measure the outcome, and *outcome parameters*, which are features of the underlying biological process under consideration and may vary from individual to individual. A diagram of an N-of-1 block design and our model of how treatment effects vary over time is shown in Figure 1.

### Case Study: Optimizing Study Design

Simulation allows us to investigate the impact of subtle design choices on the likelihood of study success. To illustrate this, we simulated a study of 2 different blood pressure medications and their impact on systolic blood pressure, similar to the data shown in [5] (see the Methods section for details). The study parameters, underlying (unobserved) data, and observed data are shown in Figure 1. The results of several hundred simulations of this study are shown in Figure 2. We used one of the standard N-of-1 regression models outlined in [6] and [11] to estimate treatment effect and obtain an associated *P* value.

In Figure 2, we see that the ordering of treatment periods has a strong effect on both statistical power and effect size estimates. On the basis of these 50 simulations, when treatments are administered in the order 1 2 1 2, power (at a standard 5% significance level) is 0.62, for 1 2 2 1 it is 0.82, for 2 1 1 2 it is 1.00, and for 2 1 2 1 it is 0.98. The median effect size estimate is also impacted by treatment ordering: for 1 2 1 2 it is 5.8, for 1 2 2 1 it is 6.6, for 2 1 1 2 it is 11.2, and for 2 1 2 1 it is 12.0. The true effect size is 10.0. We observe lower power and diminished effect size estimates for treatment orderings 1 2 1 2 and 1 2 2 1 relative to 2 1 1 2 and 2 1 2 1 as Treatment 1 takes longer to reach its full effect than Treatment 2, and the patient starts at a relatively high baseline (systolic blood pressure=160); therefore, when it is administered first, Treatment 1 never attains its full effect during the first treatment period before the transition to Treatment 2 takes place.

In Figure 2, we see the effect of sampling frequency on study power. Increasing the sampling frequency causes power to increase but only to a point. On the basis of these 50 simulations, when only 1 sample is taken at the end of each treatment period (sampling interval of 30 days), which is the most common approach to analyzing N-of-1 studies [6,11], power is only 0.14. Sampling every day (sampling interval of 1 day) yields a power of 0.84; sampling every 2 days yields a power of 0.74, every 5 days yields a power of 0.76, every 10 days yields a power of 0.56, and every 15 days yields a power of 0.50. On the basis of these results, it appears that sampling every 2 or 5 days could substantially reduce patient burden while causing only a modest reduction in power.

Figure 2 shows the effect of treatment period length, keeping the total number of blocks fixed at 2 and the sampling rate fixed at 1 sample per day. On the basis of these 50 simulations, when the treatment period length is 2 days, power is 0.18 and the mean effect size estimate is –1.5. For a period length of 5 days, power is 0.54 and the mean effect size is 3.1. For a period length of 15 days, power is 0.44 and the mean effect size is 9.7. For a period length of 30 days, power is 0.94 and the mean effect size is 10.2. For period lengths of 40, 60, and 120 days, power and mean effect sizes are 0.92 and 8.3, 0.98 and 9.7, and 0.96 and 10.6, respectively. This indicates that for a period length of 30 days, one obtains approximately as accurate an effect estimate as a period length of 60 days while shrinking the total study duration from 240 to 120 days. Period lengths that are too long run the risk of higher variance in estimates because of baseline drift, as we see with a period length of 120 days in Figure 2.

Finally, Figure 2 shows the effect of different block designs for a study of fixed length (120 days). On the basis of these 50 simulations, power for 1, 2, 3, 4, 5, and 6 blocks is 0.74, 0.86, 0.78, 0.84, 0.74, and 0.60, respectively. Mean and standard deviation of the effect size estimates are 9.7 (5.8), 9.8 (3.8), 8.7 (3.6), 8.3 (2.9), 7.0 (2.5), and 6.6 (1.8), respectively. Using 2-4 blocks appears to be the best approach, as this reduces variance in the effect size estimate relative to a single-block study. Adding more than 4 blocks increases the impact of wash-in/carryover effects on the estimate, which deviates further from its true value of 10 with each additional block.

### Case Study: Evaluating Analysis Protocols

Simulation can also help us evaluate the likely success of new analysis protocols and decision criteria for N-of-1 studies. We simulated a previously published study [13] in which the outcome was a “diary score” on a scale of 0 to 6, with 0 representing “no complaints at all” and 6 representing “unbearable complaints.” The study design used 5 blocks, each with 2 treatment periods; only data from the last week of each treatment period were analyzed.

In this paper, the data were analyzed as follows: the researchers took differences in median diary scores between NSAID and paracetamol treatment periods in each block and then calculated the number of treatment blocks for which the NSAID score was at least one point lower than the paracetamol score for the patient’s main complaint. An NSAID was recommended if this was true in at least 4 out of 5 blocks. We refer to this method as *median differencing* from now on.

We compared median differencing to the same regression model used in the previous section [11]. Simulations show that median differencing is much more conservative in recommending an NSAID than a standard regression model trained on the same data (Figure 3). For a true effect difference of size 2 (NSAID reduces pain by 2 points relative to paracetamol), median differencing will only recommend an NSAID, on average, 61% of the time, compared with 100% of the time for the regression model. In addition, median differencing will recommend an NSAID more frequently in cases where the diary score on paracetamol is already low (the patient is not in much pain); when the score is high, it becomes harder for it to detect an effect. For a patient with a paracetamol diary score of 6 (the maximum possible pain), if the NSAID reduces the diary score to 4, median differencing will only recommend an NSAID 30% of the time, as opposed to 100% of the time for the regression model. The difference between the models is even more pronounced when the NSAID only reduces the pain score by 1; in that case, median differencing will only recommend an NSAID, on average, 7% of the time, as opposed to 92% of the time for the regression model.

### Design Considerations for N-of-1 Studies

Figure 4 shows the results of a set of simulations on the basis of *best-case scenarios* — no variation in parameters other than those under investigation, as well as instantaneous treatment effects (ie, no carryover effects). The technical details of the simulations can be found in the Methods section. All of the graphs in Figure 4 relate the study design parameters to (1) statistical power—the ability to detect a treatment effect difference if it exists, and (2) the accuracy of the effect size estimates produced by the model. All compare a single treatment against placebo.

In Figures 4a and 4b, observation noise (*σ*_o_) is fixed at 1.0, with no process noise or baseline drift. As a result, “effect size really describes a signal-to-noise ratio and is treatment and instrument agnostic." We observe that this ratio impacts power but not the accuracy of the effect estimate (Figure 4).

In Figure 4a, we see that for effect sizes of 0.1, 0.2, and 0.3, more than 100 samples per treatment are needed to obtain a power of 0.8 (at a standard 5% significance level). For an effect size of 0.4, at least 100 samples per treatment are needed. For effect sizes of 0.5, 0.6, 0.7, 0.8, 0.9, and 1.0, the numbers of samples per treatment needed to attain a power of 0.8 are approximately 65, 45, 35, 26, 21, and 18, respectively. Even more samples will be needed under real experimental conditions where process noise, baseline drift, and carryover effects all play a role. This indicates that unless the effect size is very high relative to the observation noise, N-of-1 studies using only a few blocks, with a single sample taken per block (the traditional approach to analyzing N-of-1 studies), will be vastly underpowered.

A separate consideration is the error in the effect size estimate, which declines monotonically with the number of samples. In Figure 4b, we see that to obtain an estimate within 0.2 *σ*_o_ of the true estimate, at least 30 samples per treatment are needed; to reach 0.1 *σ*_o_, over 100 samples per treatment are needed.

Figure 4c shows the impact of process noise on the number of samples needed to attain a power of ≥0.8 at a 5% significance level in the absence of observation noise and baseline drift. In this figure, the intersample interval is fixed at 1 sample/time unit and the process noise is defined relative to that; *σ*_p_=1.0 indicates that if no treatment effect were present, the variance of the Wiener process underlying the process noise would be 1 outcome unit/time unit. For an effect size of 0.5 and *σ*_p_=0.0, 0.4, 0.8, 1.2, 1.6, 2.0, the numbers of samples per treatment needed to obtain a power of 0.8 are 61, 76, 89, 111, 135, and 176, respectively. For an effect size of 1.0, the numbers of samples per treatment needed are 20, 24, 28, 34, 43, and 53, respectively. Regardless of effect size, increasing the process noise from 1.0 to 2.0 roughly doubles the number of samples it takes to attain a power of 0.8. However, the effect is nonlinear; below *σ*_p_≈1.0, the number of samples needed flattens out in the absence of other sources of noise.

In Figure 4d, we see the impact on study outcome of individual sensitivity to treatment. The lower the value of the treatment sensitivity parameter (α) is, the less effect changes in treatment have on the outcome relative to random fluctuations caused by process noise. We see this when we contrast the effect of increased process noise on the minimum samples required to attain a power of 0.8 at a significance level of 5% under conditions of low treatment sensitivity (α=0.1) and high treatment sensitivity (α=10.0). For *σ*_p_=0.0, 0.4, 0.8, 1.2, 1.6, 2.0 and α=0.1, the numbers of samples per treatment required are 36, 64, 110, 174, 228, and 250, respectively. For α=10.0, the numbers of samples required are only 20, 23, 28, 34, 42, and 53, respectively.

Finally, Figure 4e shows us why we bother to have blocks at all: to guard against baseline drift. The figure shows what happens in a study of a total length of 240 days when block designs incorporating 1, 2, 3, or 4 blocks are used. As baseline drift increases (holding process and observation noise constant at *σ*_p_= *σ*_o_=0.0), the effect size estimate provided by the model increasingly deviates from its true value. This effect is most pronounced in studies with only a single block and decreases as the number of blocks increases. For example, for only 1 block, with *σ*_b_=0.00, 0.09, 0.18, 0.27, 0.37, and 0.46, the average deviation of the effect size estimate from the true value is 0.21, 0.33, 0.54, 0.77, 1.01, and 1.26, respectively. However, with 4 blocks, with the same progression of *σ*_b_ values, the average deviation of the effect size estimate is 0.21, 0.22, 0.25, 0.28, 0.32, and 0.37, respectively.

## Discussion

### Summary of the Paper

We have developed a stochastic time-series model that simulates an N-of-1 study, facilitating rapid optimization of N-of-1 study designs and increasing the likelihood of study success while minimizing participant burden. We have used this model to evaluate 2 case studies, showing how the number of treatment blocks, ordering of treatments within blocks, duration of each treatment, sampling frequency, and study analysis protocol affect our ability to detect true differences in treatment efficacy. Our simulation software is available on GitHub as described in the Methods section.

### Recommendations for the Design of N-of-1 Studies

An N-of-1 study should have as many blocks as possible to avoid baseline drift (Figure 4). If no wash-in or carryover effects are present, a single sample should be taken at the end of each of *JN* different treatment periods, where *N* is the number of blocks and *J* the number of treatments; *N* should be made as high as possible; each block should be made as short as possible. However, in practice, the number of blocks we can use in a study is bounded by the dangers of administering different treatments in rapid succession, the time it takes treatments to ramp up to their full effects (“run-in”: Table 1), the time it takes them to stop working when they are discontinued (“wash-out”: Table 1), and participant patience.

It is important to consider the fact that most N-of-1 studies of reasonable length and reasonable sampling frequency will be underpowered unless the difference in treatment effects is at least on the order of the standard deviation of the observation noise (Figure 4). The goal, perhaps obvious, should be to measure the outcome with as little noise as possible and at as high a frequency as possible, and/or to continue the study until enough samples are obtained to ensure that the effect will be detected if it is there.

Finally, it is important to remember the difference between power and accuracy. Just because a statistically significant difference in treatment effects is detected, it does not mean that the quantitative estimate of *E*_*2*_*−E*_*1*_ reported by the model is accurate. Even when a study is sufficiently powered, the effect size estimate will almost always improve with the addition of more samples.

Beyond these general statements, our main recommendation for N-of-1 study designers is to simulate the study. We can see from Figures 4c and d that process noise and individual sensitivity to treatment can have a dramatic impact on the number of samples needed to adequately power a study, especially if the effect size is small. The choice of analysis method can also have a substantial impact on study outcome and treatment recommendations (Figure 3); therefore, it is important to compare novel analysis methods to the standard models provided by the AHRQ and others [6,11]. Simulations can help in both cases.

### Modeling Different Outcome Types

Most of our analyses in this paper concerned a continuous (or near-continuous) random variable, such as blood pressure or heart rate. However, many N-of-1 trials examine outcomes that are better modeled as counts, proportions, binary random variables (yes/no), or discrete bounded scores (such as surveys). Studies with these outcome types can be simulated by transforming the output of the stochastic differential equation model using a set of transformations similar to those for generalized linear models (see Table 2). We used one such transformation to discretize the scores for the pain management case study.

### Sources of Treatment and Instrument Parameters

By far, the strongest drawback to the simulation approach is the difficulty associated with identifying reasonable simulation parameters, especially in cases where the outcome is not a continuous value (see Table 2).

Some parameters have relatively clear interpretations and can be found by looking at the known characteristics of treatments and instruments. For example, in the case of a continuous-valued outcome, we can think of the treatment effect, *X*_*j*_*(t)*, as the treatment’s maximum impact—at each point in time—on the outcome in the absence of any noise, in a population of people exactly like the one who is undergoing the study. The treatment effect is governed by 3 parameters: *τ*_*j*_, the time constant of “wash-in” for that treatment, *γ*_*j*_, the time constant of “wash-out”, and *E*_*j*_, the asymptotic effect size (the change from baseline that the person would experience in the long run was he/she to continue on this treatment). In the case of a pharmaceutical intervention, these are important parameters that have probably been estimated in earlier clinical trials and used to guide dosages, dosing frequencies, etc. Similarly, reasonable values for *σ*_o_ can often be obtained from technical specifications of whatever instrument is used to monitor the outcome.

The emerging field of mobile health may provide some help in estimating parameters like *σ*_p_ and *σ*_b_, which are properties of an outcome and its natural variation over time [14]. As we begin to monitor patients longitudinally with increasingly higher resolution, our quantitative understanding of long- and short-term variation in biological processes will naturally increase. However, in simulations at present, we recommend experimenting with varying parameter scales and examining raw plots of the data to see if the level of noise produced by the model is reasonable. It may also make sense to test ranges of *α*, *σ*_*b*_, and *σ*_*p*_ and examine plots like those shown in Figure 4 to assess the effect of these parameter choices on statistical models.

### Study Limitations and Future Work

This study fits simulated data with a simple regression model recommended by the AHRQ, but the data themselves are simulated using a more realistic model. A natural next step would be to use the full simulation model as the basis for fitting data. Future versions of our software will allow users to fit data using the AHRQ model and the full time-series model in a Bayesian framework, which infers the model parameters using posterior probability distributions given the data rather than point estimates [15,16]. Thus, uncertainty is an inherent part of the model. This will provide a basis for directly comparing the performance of the full time-series model against the simple AHRQ model for making treatment recommendations. In addition, posterior parameter distributions inferred from real data can be used to generate more realistic simulated data. This will be especially useful for studies with discrete outcomes, where the linkage between model parameters and outcome data is more difficult to interpret. Another advantage of a Bayesian parameter estimation approach is that it allows parameter estimates for N-of-1 studies to be continually updated as more individuals undergo the same study, creating a system that learns from past data to adapt the design of future studies.

One important limitation of our model is that although it incorporates multiple sources of noise, it ignores more structured sources of outcome variation (eg, variation in heart rate does not principally happen stochastically with time, but the heart rate does show structured change across hours, days, and ovulatory cycles). It is also possible that long-term seasonal, day of week, and time of day effects can influence the outcome of N-of-1 studies. Future versions of our model may incorporate parameters for these effects and fit them using methods akin to those of Prophet [17] or other Bayesian time-series models. In the meantime, users can address these issues by manually adding known sources of variation to the baseline drift term or by choosing outcome parameters that “average out” known sources of variation (eg “heart rate daily mean”).

In general, the development of realistic simulations of N-of-1 studies is an ongoing process. We believe that simulation will prove crucial as N-of-1 studies enter mainstream clinical practice, especially in the realm of precision medicine, and we hope that our model will inspire others to adopt N-of-1 studies as a tool in their own research.

## Abbreviations

AHRQ: Agency for Healthcare Research and Quality
NSAID: nonsteroidal anti-inflammatory drug
LOESS: locally-estimated scatterplot smoothing
